# Supplementary material for: Nutrimedia: A novel web-based resource for the general public that evaluates the veracity of nutrition claims using the GRADE approach
Source: PLoS One. 2020 Apr 30;15(4):e0232393. doi: 10.1371/journal.pone.0232393 (PMC7192410; doi:10.1371/journal.pone.0232393)
Supplement: S1 Table — (DOCX) [file pone.0232393.s001.docx]

## S1 Table. Resources and search strategies

| **Type of document** | **Resource** | **Search strategy** |
| --- | --- | --- |
| **Clinical practice guidelines** | MEDLINE (PubMed) | Under “Article types” in the filters menu, we selected “Practice Guideline” and “Guideline” |
|  | Guidelines International Network (if not relevant studies were found in PubMed) |  |
| **Systematic reviews** | MEDLINE (PubMed) | Under “Article types” in the filters menu, we selected “Systematic Reviews” |
|  | Cochrane Database of Systematic Reviews |  |
| **Primary studies** | MEDLINE (PubMed) | We used “Clinical Queries” |
