# Supplementary material for: Nutrimedia: A novel web-based resource for the general public that evaluates the veracity of nutrition claims using the GRADE approach
Source: PLoS One. 2020 Apr 30;15(4):e0232393. doi: 10.1371/journal.pone.0232393 (PMC7192410; doi:10.1371/journal.pone.0232393)
Supplement: S2 Table — (DOCX) [file pone.0232393.s002.docx]

## S2 Table. Key reporting aspects of the evidence

| **Clinical practice guidelines** | **Systematic reviews** | **Primary studies** |
| --- | --- | --- |
| **Objective(s)** | **Objective(s)** | **Objective(s)** |
| **Methods**  - Search strategy  - Evaluation of the certainty of the evidence  - Recommendations | **Methods**  - Methodology used (with or without meta-analysis)  - Eligibility criteria of studies  - Search strategy  - Evaluation of the certainty of the evidence | **Methods**  - Eligibility criteria  - Type of study  - Evaluation of the internal validity  - Evaluation of the external validity |
| **Main findings**  - Recommendations  - Strength and direction of recommendations | **Main findings**  - Results of meta-analysis  - Risk of bias | **Main findings**  - Absolute and relative effects (RR, OR, or HR) |
| **Conclusion(s)** | **Conclusion(s)** | **Conclusion(s)** |
| **Resource**  RIGHT checklist | **Resource**  PRISMA checklist | **Resource**  CONSORT checklist, STROBE-NUT checklist |
