# Supplementary material for: Nutrimedia: A novel web-based resource for the general public that evaluates the veracity of nutrition claims using the GRADE approach
Source: PLoS One. 2020 Apr 30;15(4):e0232393. doi: 10.1371/journal.pone.0232393 (PMC7192410; doi:10.1371/journal.pone.0232393)
Supplement: S3 Table — (DOCX) [file pone.0232393.s003.docx]

## S3 Table. Standardised statements about effect according to the GRADE approach^1^

|  | **Important benefit/harm** | **Less important benefit/harm** | **No important benefit/harm** |
| --- | --- | --- | --- |
| **High certainty evidence** | [Intervention] improves/reduces [outcome] (high quality / certainty evidence) | [Intervention] slightly improves/reduces [outcome] (high quality / certainty evidence) | [Intervention] makes little or no difference to [outcome] (high quality / certainty evidence) |
| **Moderate certainty evidence** | [Intervention] probably improves/reduces [outcome] (moderate quality / certainty evidence) | [Intervention] probably slightly improves/reduces / probably leads to slightly better/worse [outcome] (moderate quality / certainty evidence) | [Intervention] probably makes little or no difference to [outcome] (moderate quality / certainty evidence) |
| **Low certainty evidence** | [Intervention] may improve/reduce [outcome] (low quality / certainty evidence) | [Intervention] may slightly improve/reduce [outcome] (low quality / certainty evidence) | [Intervention] may make little or no difference to [outcome] (low quality / certainty evidence) |
| **Very low certainty evidence** | We are uncertain whether [intervention] improves/reduces [outcome] as the quality / certainty of the evidence has been assessed as very low | | |
| **No studies** | No studies were found that looked at [outcome] | | |

^1^Table taken from Cochrane Norway. How to write a plain language summary of a Cochrane intervention review. Appendix 1: Table of standardised statements about effect. June 2016 [cited 2019 Oct 25]. In: Cochrane Norway website [Internet]. Available from: http://www.cochrane.no/sites/cochrane.no/files/public/uploads/How to write a Cochrane PLS 9th June 2016.pdf
