# Supplementary material for: Nutrimedia: A novel web-based resource for the general public that evaluates the veracity of nutrition claims using the GRADE approach
Source: PLoS One. 2020 Apr 30;15(4):e0232393. doi: 10.1371/journal.pone.0232393 (PMC7192410; doi:10.1371/journal.pone.0232393)
Supplement: S4 Table — An example of a scientific evaluation of a nutrition claim. (DOCX) [file pone.0232393.s004.docx]

## S4 Table. Text box. An example of a scientific evaluation of a nutrition claim

| For the claim “Is meat carcinogenic?” we formulated the structured clinical question as “Does meat consumption increase the risk of cancer in the adult population?”.  We assessed the evidence related with this enquiry from the public from two recent and adequate quality SRs; the first on meat and cancer and the second on meat and colorectal cancer. The authors of the SR concerning the association between dietary patterns (high-meat versus plant-based dietary patterns) and cancer risk conducted a systematic search on PubMed and EMBASE databases and used the Newcastle-Ottawa Quality Assessment Scale to assess the quality of each prospective study included. The authors of an SR regarding meat and risk of colorectal cancer also conducted a systematic search in two databases (PubMed and EMBASE) and evaluated the risk of bias of the studies. In addition, the authors applied a NutriGrade scoring system to evaluate the trustworthiness of meta-evidence.  We considered the certainty of the available evidence regarding red meat and the risk of colorectal cancer as low because we observed inconsistency between results from some prospective studies; however, we observed the presence of a dose-response gradient. The statement about this was: “habitual red meat consumption may increase the risk of colorectal cancer”. For processed meat and the risk of colorectal cancer, we considered the certainty of the available evidence as moderate. Here we also observed the presence of a dose-response gradient. The statement about this was: “the habitual processed meat consumption probably increases the risk of colorectal cancer”. For no processed meat, we are uncertain whether a diet high in meat increases the risk of cancer as we assessed the quality of the evidence has been assessed as very low due to inconsistency and publication bias.  With these results, we deemed the overall certainty of the evidence as low and we considered the veracity of this claim possibly true. |
| --- |
